# Knowledge and practice of breast self-examination and associated factors among women with breast cancer in Kabul, Afghanistan

**DOI:** 10.1371/journal.pone.0335460

**Published:** 2025-10-24

**Authors:** Mohadeseh Ahmadi, Arash Nemat, Rohullah Sakhi, Atefa Ahmadi, Mursal Massoud, Elaha Ebadi, Mashhodullah Zahid, Arezu Nasrati, Raihana Amiri

**Affiliations:** 1 Faculty of Medicine, School of Population and Public Health, University of British Columbia, Vancouver, Canada; 2 Faculty of Global Public Health, Karolinska Institute, Stockholm, Sweden; 3 Faculty of Public Health, Kabul University of Medical Sciences, Kabul, Afghanistan; 4 Faculty of Allied Health Sciences, Kabul University of Medical Sciences, Kabul, Afghanistan; 5 Premier Urgence, Aide Medical International, Kabul, Afghanistan; 6 Elaha Ebadi, Health Specialist, Deutsche Gesellschaft fur Internationale Zusammenarbeit, Kabul, Afghanistan; Kandahar University, Faculty of Medicine, AFGHANISTAN

## Abstract

**Background:**

Breast cancer is the leading cause of cancer-related mortality among women worldwide, and it has poor prognosis if diagnosed at late stages. Common breast cancer detection methods include mammography, clinical breast exams (CBE), and breast self-examination (BSE). Breast self-examination is the most cost-effective strategy for early detection in low- and middle-income countries.

**Objective:**

To evaluate the knowledge and practice of breast self-examination, along with associated factors, among women with breast cancer visiting Ali Abad Teaching Hospital in Kabul, Afghanistan in 2025.

**Methods:**

This cross-sectional study was conducted among 290 Afghan women aged 20–80 who were either currently or previously admitted to the Oncology department of Ali Abad Teaching Hospital for regular follow-ups or treatment. Data was collected using an interviewer-administered questionnaire between January and February 2025. Chi-square tests were conducted to examine the associations between BSE knowledge, BSE practice, and potential explanatory factors. Those that showed significant associations in the bivariate analyses were considered potential confounders and included in multivariable logistics regression analysis.

**Results:**

The mean age of participants was 42.9 ± 14.7. Majority of the participants were illiterate (83.8%) and unemployed (95.9%). Women with education of secondary level or higher were more likely to practice BSE than those who were illiterate (AOR: 3.65, 95% CI: 1.06–12.76). Participants with good knowledge level were more likely to practice BSE than those who had a poor knowledge of BSE (AOR: 5.28, 95% CI: 2.45–12.48). In addition, women who had heard of BSE were more likely to practice it compared to those who had not (AOR: 4.31, 95% CI: 1.37–19.25).

**Conclusions:**

In this study, education, knowledge score, and awareness of BSE (i.e., having heard of BSE) were selected as important predictors for practice of BSE via both bivariate and multivariate logistic regression analysis. While about 50% of participants demonstrated good knowledge of BSE, only 18% were practicing it, and among those who did, only about 30% were performing it at the right time and frequency. These findings highlight the importance of educational programs with an aim to increase breast cancer awareness among women in Afghanistan, and to promote breast self-examination as a low-cost, accessible tool for early detection – helping to alleviate cancer burden in the country.

## Background

Breast cancer (BC) is the most common cancer and the leading cause of cancer-related mortality among women worldwide [1]. In 2022, 2.3 million cases of BC and 670,000 related deaths were reported globally [1]. In Afghanistan, breast cancer is one of the five top commonly diagnosed cancers in women, with 3,173 confirmed cases and 1,783 deaths reported in 2020, accounting for 26% of newly diagnosed cancers in women according to the Global Cancer 2020 statistics [2,3]. According to WHO estimates from 2012, Afghanistan has the highest number of breast cancer cases compared to its neighboring countries – excluding Pakistan – including Iran, China, India, Uzbekistan, Tajikistan, and Turkmenistan [4]. A recent commentary article has demonstrated potential challenges in cancer care in the current sociopolitical context of Afghanistan [5].

Breast cancer is a preventable disease, with age as an important risk factor – particularly affecting women in their most productive years. While the disease is prevalent globally, more cases are identified in developing countries among women aged of 15–49 [6]. Other risk factors include modifiable lifestyle choices such as obesity, physical activity (PA), food choices, smoking, and alcohol use [4,7–12]. Treatment efficiencies vary significantly between high and low- and middle-income countries [1]. In high-income countries, advances in early detection and treatment have greatly improved survival rates, with 90% of women surviving for at least five years post-diagnosis [1]. Survival rates in low-income countries range from 40 to 66%, primarily due to inadequate treatment options and limited access to early detection [5,13]. This inequality is concerning.

In Afghanistan, as in many low-resource settings, breast cancer treatment options are scarce. The healthcare system is heavily dependent on international aid to address issues related to and manage breast cancer [2]. While it is possible to continue relying on comprehensive treatment plans provided by high-income countries, the situation in Afghanistan is far more challenging, particularly due to underdeveloped healthcare infrastructure, shortage of medical supplies, and unskilled personnel [5]. As a result, many patients need to seek treatment in neighboring countries like Pakistan and Iran [5]. The lack of domestic treatment and infrastructure, combined with a healthcare system mainly focused on communicable disease and reproductive health, underscore the need for effective prevention strategies [13]. Furthermore, while Afghanistan has a national cancer policy, it may be outdated, and cancer registries remains limited [4]. The only known cancer registry operates in Kabul at Jamhuriat Hospital; however, national data collection continues to be a challenge.

Given the limitations in Afghanistan’s healthcare system, the most viable approach to reduce breast cancer burden is prevention through early detection and prompt diagnosis [6,7,14]. Screening practices are essential for rapid diagnosis – improving the likelihood of early treatment, and significantly reducing mortality risk [4]. Breast cancer is almost completely curable when detected early and by seeking medical care in the course of the disease. The three major methods to detect breast cancer are mammography, clinical breast exams (CBE), and breast self-examination (BSE). In resource-limited settings, where mammography and clinical breast exams are difficult to implement, BSE becomes the most cost-effective method for early detection [6]. BSE is a process whereby women examine their breast for any swelling or other abnormalities [6]. With proper training and counselling from healthcare professionals, combined with regular practice, women can increase their awareness of breast health and seek medical care earlier, when treatment is most effective [6,7,15,16].

Studies suggest women’s knowledge about breast cancer risk factors and early warning signs contribute to their likelihood of accessing medical care or early intervention [12,17,18]. Despite the benefits of BSE, knowledge and practice among Afghan women remain relatively low, largely due to lack of education and cultural barriers. Therefore, this study is designed to assess the knowledge, practice, and associated factors of BSE among Afghan women aged 20–80, accessing care at the Oncology department of Ali Abad Teaching Hospital in Kabul city.

## Methods

### Study design and setting

This cross-sectional study was conducted on 290 female participants aged 20–80 receiving care at Ali Abad Teaching Hospital in Kabul between January 15 and February 15, 2025. The hospital is one of the largest research, teaching, and healthcare service centers in Afghanistan, affiliated with Kabul University of Medical Sciences. This medical center recently established a 400-bed cancer treatment section, which is now operational. Kabul, the capital city of Afghanistan, with a population of 4.2 million people, is located in the eastern part of Afghanistan [4].

### Inclusion and exclusion criteria

We included individuals that met the following criteria: currently or previously admitted to the Oncology department of Ali Abad Teaching Hospital for regular follow-ups or treatments; women aged 20–80; without mental health conditions or other disorders that may impair their ability to understand the questionnaire; and women willing to participate in the study.

### Sample size determination

**Fig 1:**
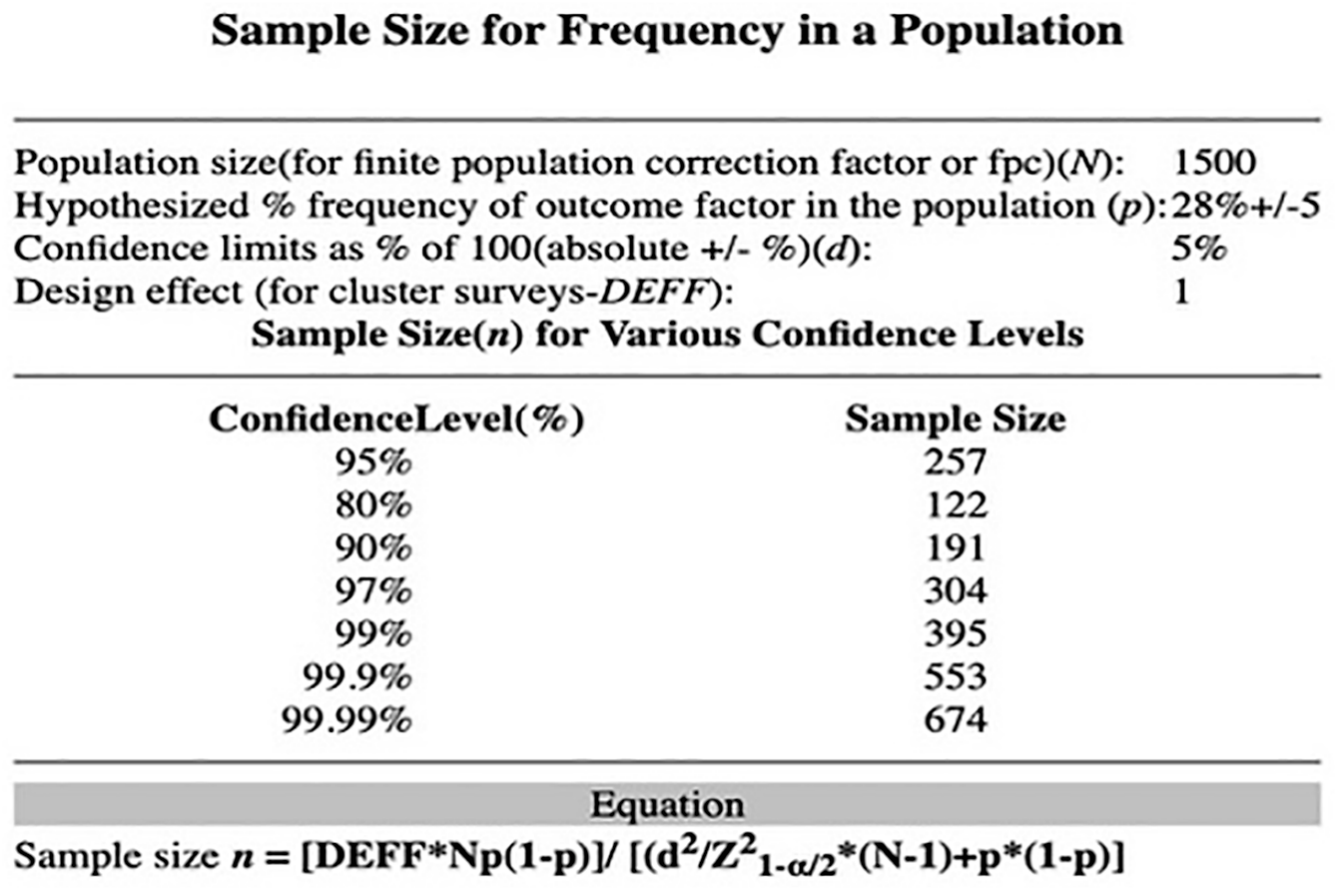
Data source: OpenEpi. The table provides sample size calculation based on population size of 1500, expected frequency of 28%, and a 95% confidence interval.

The sample size was calculated using the anticipated 28% frequency of breast self-examination practice in the general female population in Afghanistan [4], due to the absence of prior estimates specifically among women diagnosed with breast cancer. Using a 5% maximum allowable error, and a 95% confidence interval, the formula (z2p[1‑p])/d2 was applied, where z = 1.96, P = 0.28 and d = 0.05. This yielded a sample size of 257, however with a non-response rate of 10%, the final sample size obtained was 282, rounded to 290 (Fig 1).

### Sampling procedure

The sampling procedure followed a convenient sampling approach, recruiting women who were readily available at the facility. This strategy was adopted due to the practical challenges of conducting research in a war-torn country and the limited feasibility of accessing a randomized population in the region.

### Study instruments

There were 10 questions used to assess participants’ knowledge of BSE. The indicators covered key areas such as their understanding of the importance of BSE, the techniques they employ during self-examination, and the signs they should be looking for. A score was calculated for each participant based on the number of questions correctly answered in this section, with correct answers given a score of “1” and incorrect responses given a score of “0”. Mean knowledge score was calculated. Participants with score below mean were classified as poor knowledge and those with scores above the mean or equal to the mean were classified as good knowledge.

Finally, participants’ actual practice of BSE was determined from binary outcome variable (yes, no); other questions were designed to also explore participants’ motivation for practicing BSE, and the age in which they started the practice. Good practice refers to those who performed BSE at least once per month – ideally one week after menstruation for premenopausal women, or on a consistent monthly date for postmenopausal women [19], and poor practice refers to performing BSE at incorrect times in the cycle [19].

### Data collection procedure and quality assurance

For quantitative data collection, participants were required to complete a detailed, pre-designed questionnaire adapted from the literature [4,7,8], which was translated into the local languages (Dari and Pashto) and back translated into English. The questionnaire consists of three sections: sociodemographic data, knowledge and practice of BSE questions. Sociodemographic characteristics include age, marital status (married and unmarried), education level (illiterate, primary school, secondary or university graduate), Body Mass Index (BMI) (underweight, normal weight, overweight, obese), occupation (employed and unemployed), distance from home to hospital ≤5 km, > 5km), and number of children (≤2, > 2). Efforts were made to measure or obtain any missing variable where feasible. For example, weight, height, waist circumference (WC), and BMI were calculated for all participants according to standard procedures [20].

A pilot study was conducted to assess the feasibility of the data collection procedure, test the clarity and understandability of the questionnaires, estimate the required time to complete the questionnaire, and identify potential challenges. Feedback from participants was used to make necessary revisions to the questionnaire. Data were collected through self-administered questionnaire for literate participants, and through face-to-face interviews for illiterate participants, conducted in a private room to ensure confidentiality. The co-investigator (RS) coordinated the interview process and reviewed questionnaires to ensure completeness of the data.

### Statistical analysis

The data analysis was conducted using R statistical computing environment. Descriptive statistics summarized demographic characteristics, with means and standard deviations for continuous variables and frequency distributions for nominal variables. The Chi-square test was used to examine relationships between variables, with p-value of <0.05 considered statistically significant. Bivariate analyses were used to assess the relationship between explanatory variables and the outcome variable.

Bivariate logistic regression models were developed to explore associations between explanatory variables and BSE practices. Variables with p < 0.05 – including education level, BSE knowledge, and disease awareness – were included in the multivariable model. Further, variables such as age, occupation, marital status, BMI, number of children, and distance from home to hospital that did not reach statistical significance in bivariate analysis were also retained based on theoretical relevance. In the multivariable logistic regressions, we estimated adjusted odds ratio (AORs) and 95% confidence intervals to understand the impact of these factors on BSE practice.

### Ethical consideration

The ethical approval was obtained from Kabul University of Medical Sciences and the Review Board of Ali Abad Teaching Hospital in October 2024. Participation in the study was voluntarily, and all participants were informed that they could withdraw at any time without consequences. Both verbal and written consents were obtained before data collection. Data was anonymized, and participants were assigned a unique identification number for use during analysis.

### Inclusivity in global research

Additional information regarding the ethical, cultural, and scientific considerations specific to inclusivity in global research is included in the Supporting Information (SX Checklist).

## Results

### Socio-demographic characteristics

Out of 337 women with breast cancer who completed the questionnaire, 28 were excluded due to missing data on key explanatory or outcome variables, and another 19 were excluded for being under the age of 19 years. In the final analytical population (Table 1), the majority of the participants were of the age group of 50 years and older (mean age: 42.9, SD: 14.7), were married (83.8%), unemployed (95.9%), illiterate (83.8%), and had more than two children (80.3%). Notably, 50.3% and 68.6% of the participants had healthy BMI and lived more than 5 km from the hospital, respectively.

**Table 1:** Sociodemographic characteristics of the study participants (n = 290).

Characteristics	Frequency	Percentage
**Age (years), mean ± SD**	42.9 ± 14.7	–
**Age (in years)**		
20-29	60	20.7
30-39	54	18.6
40-49	83	28.6
50+	93	32.1
**Marital status**		
Married	243	83.8
Unmarried	47	16.2
**Occupation**		
Employed	12	4.1
Unemployed	278	95.9
**Education level**		
Illiterate	243	83.8
Primary	17	5.9
Secondary & higher education	30	10.3
**Body mass index (BMI)**		
Underweight	82	28.3
Healthy	146	50.3
Overweight	57	19.7
Obese	5	1.7
**Distance from home to hospital**		
≤5 km	91	31.4
>5 km	199	68.6
**Number of children**		
≤2 children	57	19.7
>2 children	233	80.3

### Knowledge and practice pertaining to BSE

**Fig 2:**
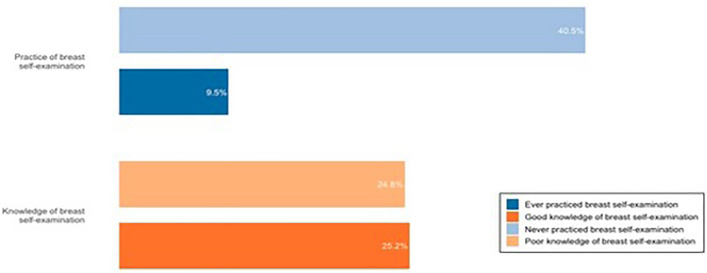
Distribution of knowledge and practice of breast self-examination among women with breast cancer (n = 290).

Out of 290 participants, 49.7% demonstrated poor knowledge of BSE (S1 Table). While only 19% reported ever practicing BSE; among them, 36.4% performed it at the right time, 34.5% started at the right age, 32.7% practiced it at the correct frequency, and 61.8% knew the correct technique. The main reasons for not practicing BSE were the absence of breast-related problems (49.8%) and lack of knowledge on how to perform BSE (29.2%). The distribution of knowledge and practice is presented in Fig 2.

### Multivariate analysis

Variables significantly associated with BSE practice in bivariate analysis (see Supplementary Tables 3S and 4S) were considered for inclusion in the multivariable logistic regression model. After adjustment for potential confounders, the strength and direction of associations of all variables remained largely consistent. Similar to bivariate logistic regression result, women education level [AOR: 3.65, 95% CI (1.06–12.76)], their awareness level of BSE [AOR: 4.31, 95% CI (1.37–19.3)], and knowledge about BSE [AOR: 5.28, 95% CI (2.45–12.48)] continued to show significant association with BSE practice. The AOR associated with these variables were slightly attenuated, but the association remained statistically significant. Other socioeconomic variables such as age, marital status, BMI, distance to the hospital, occupation, and number of children were still not significantly associated with BSE practice after adjustment. Findings of multivariate logistic regression analysis is present in detail in Table 2.

**Table 2:** Multivariable analysis of factors associated with BSE practice among women with breast cancer visiting Ali Abad Teaching Hospital.

Characteristics	COR (95% CI)	p-value	AOR (95% CI)	p-value
**Age (in years)**				
20-29	Reference	–	Reference	–
30-39	1.01 (0.39-2.63)	0.98	0.83 (0.24-2.90)	0.76
40-49	1.15 (0.50-2.73)	0.75	0.97 (0.29-3.41)	0.97
50+	0.99 (0.43-2.36)	0.99	0.97 (0.28-3.52)	0.97
**Marital status**				
Married	Reference	–	Reference	–
Unmarried	0.86 (0.35-1.87)	0.71	0.79 (0.20-2.78)	0.72
**Occupation**				
Unemployed	Reference	–	Reference	–
Employed	1.45 (0.31-5.05)	0.59	1.59 (0.24-9.06)	0.61
**Education level**				
Illiterate	Reference	–	Reference	–
Primary	1.52 (0.41-4.54)	0.49	1.93 (0.45-7.23)	0.35
Secondary & higher education	2.46 (1.04-5.55)	0.033*	3.65 (1.06-12.76)	0.04*
**Body mass index (BMI)**				
Underweight	0.87 (0.41-1.78)	0.71	1.26 (0.53-2.94)	0.59
Healthy	Reference	–	Reference	–
Overweight	1.50 (0.71-3.11)	0.28	1.40 (0.60-3.19)	0.43
Obese	3.08 (0.39-19.5)	0.23	1.45 (0.17-9.78)	0.70
**Distance from home to hospital**				
≤5 km	Reference	–	Reference	–
>5 km	0.69 (0.37-1.28)	0.23	0.93 (0.46-1.91)	0.84
**Number of children**				
≤2 children	Reference	–	Reference	–
>2 children	1.13 (0.55-2.51)	0.76	1.88 (0.54-7.20)	0.33
**Heard of BSE**				
No	Reference	–	Reference	–
Yes	7.06 (2.49-29.7)	0.0014*	4.31 (1.37-19.25)	0.03*
**Level of knowledge**				
Poor	Reference	–	Reference	–
Good	5.97 (2.98-13.1)	<0.05*	5.28 (2.45-12.48)	<0.05*

*Statistically significant at a level of p-value<0.05; COR: crude odds ratio; AOR: adjusted odds ratio; CI: confidence interval; -: reference group

## Discussion

In this study, we assessed the knowledge and practice level associated with breast self-examination among women with breast cancer in Kabul, Afghanistan. While about half of the population demonstrated good knowledge of breast self-examination, only 19% self-reported its practice. The finding highlights gap in awareness of BSE practice among women already diagnosed with breast cancer. Multivariable analysis identified BSE awareness, knowledge level, and education as significant predictors of BSE practice, while other sociodemographic factors such as age, BMI, marital status, number of children, employment status, and distance from hospital were not significantly associated.

Nealy half of the participants had poor knowledge of BSE. This finding is consistent with studies in Nepal (44%) [21], Banke District (44%) [22], Ethiopia (50%−56%) [19,23], and Ghana (44%) [24]. However, higher levels of BSE knowledge have been reported among female workers in Debre Tabor Town, Ethiopia [25], women of reproductive age in Southeast Ethiopia [7], and women in Malaysia [8]. The relatively low level of knowledge in our study may be due to low employment rate among Afghan women, lack of breast cancer awareness programs, cultural barriers, and lower education levels; only 10% of participants had attained secondary or high education, compared to 59%−84% in other studies [8,25]. These findings suggest that promoting female education and integrating health education into school curricula and adult learning programs may help improve BSE knowledge.

In this study, only 19% of participants reported practicing BSE, which aligns with low practice rates in Iran (29%) [14], Ethiopia (13%−21%) [7,23,26,27], Nepal (12%) [22], Cameroon (15%) [27], and Kuwait (21%) [28]. Higher practice rates have been observed in Northwest Ethiopia (46%) [19], Malaysia (55%) [8], and Nigeria (43%) [12]. The difference in finding between our study and studies reporting higher BSE practice may be attributable to limited BSE knowledge, cultural factors, and low education levels. Notably, even among those practicing BSE, many did not perform it correctly in relation to its frequency, timing, and technique. This gap highlights the need for not only awareness but also skill-based training and community-level education campaigns.

BSE awareness and higher knowledge levels were significantly associated with BSE practice in our study. In bivariate analyses using Chi-square and Fisher’s exact tests, no sociodemographic variables showed significant association with BSE practice, unlike other studies where age and other factors were associated with practice. This lack of association may be due to the low proportion of women practicing BSE (18%), leading to imbalance in the outcome variable and reduced statistical power within subgroups. After multivariable adjustment, however, BSE awareness and knowledge remained significant predictors, consistent with findings from Ethiopia [7], Malaysia [8], and Nigeria [9]. These results highlight the importance of targeted educational interventions to improve both awareness and practical skills for effective BSE practice.

Education level was independently associated with BSE practice after adjusting for potential confounders. Women with higher education are more likely to adopt preventive health behaviours, consistent with other studies [7–9]. Other sociodemographic factors such as employment status, age, number of children, marital status, BMI, and distance from hospital were not significantly associated in our sample, which may reflect the characteristics of women with breast cancer, who are more likely to receive counselling and regular healthcare interaction than the general population. These findings suggest that integrating BSE education into school curricula, adult literacy programs, and clinical counselling may improve BSE practice among women with lower education.

In Afghanistan, there is only one study focusing on BSE among women visiting hospitals, while the present study aims to explore the knowledge and practice of BSE among women diagnosed with breast cancer [4]. Findings from our study provide critical insight for survivorship care, emphasizing the importance of promoting BSE in a resource-limited setting where clinical follow-ups and diagnostic services are scarce. Policymakers should consider integrating BSE education into national health programs, and healthcare providers should routinely counsel women to empower self-monitoring. Future research should explore cultural and educational barriers to inform targeted, culturally appropriate awareness campaigns aimed at improving early detection.

## Strengths and limitations

This study has strengths and limitations. This study focuses on a high-risk population who have already been diagnosed with breast cancer. This approach can provide valuable insight into whether BSE was effective in early detection of the condition or whether it was neglected. Fortunately, this study is conducted in Afghanistan, which appropriately reflects the cultural and social barriers specific to Afghan women.

The present study also has several limitations. First, the observations are based in Kabul, Afghanistan, and while they offer insights relevant to the capital city, the findings may not be generalizable to rural community, where healthcare resources and access are more limited, or to other urban areas across the country. Second, the results are based on self-reported data, which may introduce recall bias, underreporting, or exaggeration, and may not accurately reflect actual BSE knowledge or practice. Third, participants were recruited using convenience sampling, which could introduce selection bias if non-responders differ meaningfully from responders. Fourth, the cross-sectional study design prevents causal inference between the factors studied and BSE knowledge or practice. In addition, some potentially important variables were not captured in our questionnaire, such as residence (urban vs. rural), household income, and family history of breast cancer. Including these variables in future studies conducted in Afghanistan could provide additional insight for policymakers and healthcare providers, particularly regarding access barriers and context-specific interventions. Further research is needed to understand whether some factors have effects beyond association.

## Conclusion

This study concludes that about half of the participants had poor knowledge of BSE, but only 19% practiced it. Among those who were practicing BSE, only about 30% knew when to perform the test, at what age to start, and how often to practice. The study also concludes that variables such as women’s education, awareness level about BSE, and knowledge levels are significant predictors of BSE practice. Findings from this study supports implementations of comprehensive BSE educational programs to increase breast cancer awareness. Our result, however, necessitate replication in different provinces of Afghanistan, particularly among the general population, and potentially using a larger sample size.

## Supporting information

S1 File Inclusivity-in-global-research-questionnaire. (DOCX)

S1 Table Knowledge about BSE among women with breast cancer visiting Ali Abad Teaching Hospital. (DOCX)

S2 Table Practice-related information on BSE among women with breast cancer visiting Ali Abad Teaching Hospital. (DOCX)

S3 Table Factors associated with BSE practice among women with breast cancer visiting Ali Abad Teaching Hospital. (DOCX)

S4 Table Association between level of BSE knowledge and practice among women with breast cancer visiting Ali Abad Teaching Hospital. (DOCX)

S1 Data Supporting Information – Data. (XLSX)
