# Supplementary material for: Knowledge and practice of breast self-examination and associated factors among women with breast cancer in Kabul, Afghanistan
Source: PLoS One. 2025 Oct 24;20(10):e0335460. doi: 10.1371/journal.pone.0335460 (PMC12551836; doi:10.1371/journal.pone.0335460)
Supplement: S1 Table — (DOCX) [file pone.0335460.s002.docx]

**Supplementary**

*Table 1S: Knowledge about BSE among women with breast cancer visiting Ali Abad Teaching Hospital*

| Questions | Frequency | % |
| --- | --- | --- |
| *Have you heard about BSE | 219 | 75.5 |
| *Changes in the shape and color of the breast are the signs of breast cancer. (Yes) | 214 | 73.8 |
| *At what age do women have to start BSE? (from age 20) | 61 | 21.0 |
| *How often should BSE be performed? (Monthly) | 73 | 25.2 |
| *Which hand do you use to examine the breast? (Use the right hand for the left breast and the left hand for the right breast) | 56 | 19.3 |
| *How do you perform BSE? (palpate with palm and three fingers) | 85 | 29.3 |
| *Lumps in the breast and around the armpit are signs of breast cancer. (Yes) | 240 | 82.8 |
| *Nipple discharge and retraction are the signs of breast cancer. (Yes) | 234 | 80.7 |
| *When should you practice BSE? (A week after menstruation) | 73 | 25.2 |
| *When examining the breast, which area do you examine? (The entire area that extends from the breast, up the breastbone area and collar area) | 108 | 37.2 |
| *When examining the breast, what type of pattern do you use? (circular) | 125 | 43.1 |
