# Supplementary material for: Knowledge and practice of breast self-examination and associated factors among women with breast cancer in Kabul, Afghanistan
Source: PLoS One. 2025 Oct 24;20(10):e0335460. doi: 10.1371/journal.pone.0335460 (PMC12551836; doi:10.1371/journal.pone.0335460)
Supplement: S2 Table — (DOCX) [file pone.0335460.s003.docx]

**Supplementary**

*Table 2S: Practice-related information on BSE among women with breast cancer visiting Ali Abad Teaching Hospital*

| Questions | Frequency | % |
| --- | --- | --- |
| **Have you ever done BSE?** |  |  |
| Yes | 55 | 19.0 |
| No | 235 | 81.0 |
| **If “Yes”, when did you perform it?** |  |  |
| During menstrual flow | 16 | 29.1 |
| A week after menstruation* | 20 | 36.4 |
| Before menstrual flow | 5 | 9.1 |
| During breastfeeding | 14 | 25.4 |
| **If “Yes”, at what age have you started BSE?** |  |  |
| From age 20* | 19 | 34.5 |
| From age 30 | 16 | 29.1 |
| From age 40 | 7 | 12.7 |
| Don’t know | 13 | 23.7 |
| **If “Yes”, how often do you practice BSE?** |  |  |
| Weekly | 24 | 43.6 |
| Monthly* | 18 | 32.7 |
| Yealy | 3 | 5.5 |
| Don’t know | 10 | 18.2 |
| **If “Yes”, which hand do you use to examine the breast? (Use the right hand for the left breast and the left hand for the right breast)** |  |  |
| Use the right hand for the left breast and the left hand for the right breast* | 7 | 12.7 |
| Use the right hand for both breasts | 17 | 30.9 |
| Use the left hand for both breasts | 7 | 12.7 |
| Use the right hand for the right breast and the left hand for the left breast | 24 | 43.7 |
| **If “Yes”, how do you perform BSE? (palpate with palm and three fingers)** |  |  |
| Palpate with one finger | 11 | 20.0 |
| Anyhow | 4 | 7.3 |
| Palpate with palm | 6 | 10.9 |
| Palpate with palm and three fingers* | 34 | 61.8 |
| **If “Yes”, when examining the breast, which area do you examine? (The entire area that extends from the breast, up the breastbone area and collar area)** |  |  |
| Axilla breast | 3 | 5.5 |
| Breast only | 10 | 18.2 |
| Breast and up the breastbone area | 13 | 23.6 |
| The entire area that extends from the breast, up the breastbone area and collar area* | 29 | 52.7 |
| **If “Yes”, when examining the breast, what type of pattern do you use? (circular)** |  |  |
| No pattern | 17 | 30.9 |
| Circular* | 37 | 67.3 |
| Wedge | 1 | 1.8 |
| Vertica strips | 0 | 0 |
| **If “Yes”, are there changes in the shape and color of the breast are the signs of breast cancer. (Yes)** |  |  |
| Yes* | 53 | 96.4 |
| No | 2 | 3.6 |
| **If “Yes”, are there nipple discharge and retraction are the signs of breast cancer. (Yes)** |  |  |
| Yes* | 54 | 98.2 |
| No | 1 | 1.8 |
| **If “Yes”, are there lumps in the breast and around the armpit are signs of breast cancer. (Yes)** |  |  |
| Yes* | 54 | 98.2 |
| No | 1 | 1.8 |
| **If “No”, what are the reasons for non-compliance? (n=233)** |  |  |
| Don’t have breast problem | 116 | 49.8 |
| Not necessary | 45 | 19.3 |
| Don’t know how to self-examine | 68 | 29.2 |
| Laziness | 4 | 1.7 |
