# Supplementary material for: Knowledge and practice of breast self-examination and associated factors among women with breast cancer in Kabul, Afghanistan
Source: PLoS One. 2025 Oct 24;20(10):e0335460. doi: 10.1371/journal.pone.0335460 (PMC12551836; doi:10.1371/journal.pone.0335460)
Supplement: S3 Table — (DOCX) [file pone.0335460.s004.docx]

**Supplementary**

*Table 3S: Factors associated with BSE practice among women with breast cancer visiting Ali Abad Teaching Hospital*

| Variables | Practice of BSE | | Test Used | P-value |
| --- | --- | --- | --- | --- |
|  | Yes | No |  |  |
|  | 55 (19.0%) | 235 (81.0%) |  |  |
| **Age (in years)** |  |  |  |  |
| 20-29 | 11 (20.0) | 49 (20.9) | Chi-square | 0.98 |
| 30-39 | 10 (18.2) | 44 (18.7) |  |  |
| 40-49 | 17 (30.9) | 66 (28.1) |  |  |
| 50+ | 17 (30.9) | 76 (32.3) |  |  |
| **Marital status** |  |  |  |  |
| Married | 47 (85.5) | 196 (83.4) | Chi-square | 0.87 |
| Unmarried | 8 (14.5) | 39 (16.6) |  |  |
| **Occupation** |  |  |  |  |
| Employed | 3 (5.5) | 9 (3.8) | Fisher’s Exact test | 0.70 |
| Unemployed | 52 (94.5) | 226 (96.2) |  |  |
| **Education level** |  |  |  |  |
| Illiterate | 41 (74.5) | 202 (86.0) | Fisher’s Exact test | 0.08 |
| Primary | 4 (7.3) | 13 (5.5) |  |  |
| Secondary & higher education | 10 (18.2) | 20 (8.5) |  |  |
| **Body mass index (BMI)** |  |  |  |  |
| Underweight | 13 (23.6) | 69 (29.4) | Fisher’s Exact test | 0.29 |
| Healthy | 26 (47.3) | 120 (51.1) |  |  |
| Overweight | 14 (25.5) | 43 (18.2) |  |  |
| Obese | 2 (3.6) | 3 (1.3) |  |  |
| **Distance from home to hospital** |  |  |  |  |
| =<5 km | 21 (38.2) | 70 (29.8) | Chi-square | 0.30 |
| >5 km | 34 (61.8) | 165 (70.2) |  |  |
| **Number of children** |  |  |  |  |
| =<2 children | 10 (18.2) | 47 (20) | Chi-square | 0.91 |
| >2 children | 45 (81.8) | 188 (80) |  |  |
| **Heard of BSE** |  |  |  |  |
| Yes | 52 (94.5) | 167 (71.1) | Chi-square | 0.0005* |
| No | 3 (5.5) | 68 (28.9) |  |  |

*Statistically significant at a level of p-value<0.05
