# Supplementary material for: Knowledge and practice of breast self-examination and associated factors among women with breast cancer in Kabul, Afghanistan
Source: PLoS One. 2025 Oct 24;20(10):e0335460. doi: 10.1371/journal.pone.0335460 (PMC12551836; doi:10.1371/journal.pone.0335460)
Supplement: S4 Table — (DOCX) [file pone.0335460.s005.docx]

**Supplementary**

*Table 4S: Association between level of BSE knowledge and practice among women with breast cancer visiting Ali Abad Teaching Hospital*

| Variables | Practice of BSE | | Chi-square | P-value |
| --- | --- | --- | --- | --- |
|  | Yes | No |  |  |
|  | 54 (18.7) | 235 (81.3) |  |  |
| **Level of knowledge** |  |  |  |  |
| Good | 45 (81.8) | 101 (43.0) | 25.4 | <0.05* |
| Poor | 10 (18.2) | 134 (57.0) |  |  |

*Statistically significant at a level of p-value<0.05
